# Long-term safety, health and mental status in men with vasectomy

**DOI:** 10.1038/s41598-018-33989-5

**Published:** 2018-10-24

**Authors:** Kai Zhao, Li Wu, Xiangbin Kong, Yaoping Chen, Honggang Li, Yiqun Gu, Xuejun Shang, Chengliang Xiong

**Affiliations:** 1 Center of Reproductive Medicine, Family Planning Research Institute, Tongji Medical College, Huazhong University of Science and Technology, Wuhan, 430030 China; 2 Wuhan Tongji Reproductive Medicine Hospital, Wuhan, China; 3 Center of Reproductive Medicine, Tongji Hospital, Huazhong University of Science and Technology, Wuhan, China; 4 National Health and Family Planning Key Laboratory of Male Reproductive Health, National Research Institute for Family Planning, Beijing, China; 5 Institute of Laboratory Medicine, Jinling Hospital, Nanjing University School of Medicine, Nanjing, Jiangsu China

## Abstract

Vasectomy is an efficient male contraceptive method, but the long-term effects of this technology in a large population are unclear. To investigate the influence of vasectomy on long-term health effects, we recruited 485 men with a vasectomy and 1940 men without vasectomy in China. After obtaining basic information from the Aging Males’ Symptoms (AMS) scale and other questionnaires, peripheral blood was drawn to assess the hormone levels, prostate specific antigen (PSA) and blood biochemistry. Using multiple linear regression analysis, these factors had no relationship with vasectomy except for four factors including the Somatic score (0.31, 0.02 and 0.61) in AMS, SF-36 score (−18.8, −32.00 and −5.60), “Role emotional” (−6.28, −10.34 and −2.22) and “Mental health” (−1.55, −3.08 and −0.02). A stratified analysis showed that with increased age, smoking and drinking, residence in township or a higher level of education, the mental health of men was worse. Vasectomy had no long-term effect on the level of sexual hormones in men, and it did not increase the level of PSA. The impact of the vasectomy on quality of life in men were mainly reflected in psychological effects, which suggests that men with vasectomy groups many benefit from professional psychological counseling.

## Introduction

Vasectomy is a male contraceptive method involving a small operation of the vas deferens. It is a simple, effective and permanent method of male contraception. Globally, approximately 5% of married couples of reproductive age depend on vasectomy as a contraceptive method^1^. In countries such as China and India, vasectomy is regarded as an extremely popular and safe method of sterilization, especially during the period of the 1960s to 1970s, even though the initially high rate of acceptance has decreased^2^. Because of the crucial role that vasectomy plays, many studies have attempted to better understand the consequences of vasectomy^3–5^.

Some studies have suggested that the intraluminal pressure of the reproductive tract can damage the testis and epididymis due to the sperm accumulation after vasectomy^6^. As a consequence, therefore, researchers assessed testosterone levels and testosterone deficient symptoms after vasectomy. The total testosterone level is usually tested in the clinic, although free testosterone is more accurate for assessment^7^. The testosterone deficiency symptoms are not specific and the questionnaires are designed to assess the symptoms. The AMS (the Aging Male’s Symptoms scale) contains seventeen questions and is used to assess testosterone deficiency symptoms, such as LOH (late onset hypogonadism)^8^.

A fatal risk factor is the risk of prostate cancer. Previously many studies that focused on assessing the relationship between prostate cancer risks and vasectomy have reported contradictory results^9–11^. Prostate symptoms are assessed by the International Prostate Symptom Score (IPSS) questionnaire^12^. In addition to testis and prostate-relevant syndromes, as far as we know, little evidence has been provided to estimate the effects on other organs, such as the metabolic illnesses. Additionally, quality of life after the vasectomy was usually ignored in previous studies. Furthermore, post-vasectomy complications have focused on physical complaints, such as post-operative pain^13,14^. However, evidence suggests that the process may also be associated with by psychological complications, including depression, irritability and somatic symptoms^15^.

However, these studies are limited. Therefore, it is thus timely to study the long-term safety and mental consequences and to recommend further research on this method of contraception. The aim of this study was to understand the effects of vasectomy over a long-term postoperative period by analyzing of the laboratory parameters and questionnaires.

## Results

### Characteristics of the subjects

The characteristics of 485 men with vasectomy and 2425 men without vasectomy are shown in Table 1. The age of the two groups is matched with a mean age of 58.3 years. There is no significant difference in weight (*P* = 0.07), BMI (*P* = 0.77) or abdomen circumference (*P* = 0.51) in the anthropometric measures. Smoking status, alcohol intake and education also showed no differences (*P* = 0.72, *P* = 0.72, *P* = 0.92). Compared to men without vasectomy, more individuals with vasectomy lived in villages (P < 0.001). The mean scores of the questionnaires including the AMS, SF-36 and IPSS were the same between the two groups but the mean score of the Beck Depression Inventory was higher in men with vasectomy(*P* = 0.01).

**Table 1:** Distribution of potential confounders among men with and without vasectomy.

	Total (N = 2425)	With vasectomy (N = 485)	Without vasectomy (N = 1940)	P value
**Age and anthropometric measures**				
Age (years)	58.3 ± 8.5	58.4 ± 8.5	58.3 ± 8.4	0.85
Height (cm)	164.2 ± 6.7	162.9 ± 6.7	164.6 ± 6.7	<0.001
Weight (kg)	64.7 ± 11.3	63.9 ± 11.6	64.9 ± 10.9	0.07
BMI (kg/m^2^)	23.9 ± 3.4	23.97 ± 3.63	23.9 ± 3.6	0.77
AC (cm)	85.4 ± 11.4	85.1 ± 11.4	85.4 ± 11.4	0.51
Smoking status - no./total (total no. (%))				0.72
Never smoked	795 (32.8)	147 (30.6)	648 (33.4)	
Former smoker	301 (12.4)	56 (11.5)	245 (12.6)	
Current smoker	1329 (54.8)	281 (57.9)	1048 (54.0)	
Alcohol intake (total no. (%))				0.72
None	717 (29.6)	132 (27.2)	585 (30.2)	
1–4 days/wk	1348 (55.6)	267 (55.1)	1081 (55.7)	
≥5 days/wk	360 (14.8)	86 (17.7)	274 (14.1)	
Education (total no. (%))				0.92
0–9 years	1006 (41.4)	214 (44.1)	792 (40.8)	
>10 years	1419 (58.6)	271 (55.9)	1148 (59.2)	
Residence no./total (total no. (%))				<0.001
township	754 (31.1)	101 (20.8)	653 (33.7)	
village	1671 (68.9)	384 (79.1)	1287 (66.3)	
Married	2239 (92.3)	455 (93.8)	1784 (91.9)	0.17
**Health status**				
AMS	30.62 ± 9.74	31.27 ± 10.16	30.45 ± 9.63	0.58
SF-36 score	539.96 ± 132.00	524.17 ± 133.02	543.90 ± 131.48	0.45
IPSS	6.50 ± 7.68	6.92 ± 7.46	6.40 ± 7.73	0.79
Score on Beck Depression Inventory	3.51 ± 4.83	3.93 ± 5.39	3.41 ± 4.67	0.01
**Hormone levels**				
TT (nmol/l)	16.16 ± 5.59	16.36 ± 6.01	16.11 ± 5.36	0.02
LH (U/l)	6.46 ± 4.76	6.78 ± 5.31	6.38 ± 4.57	0.07
SHBG (nmol/l)	49.86 ± 24.36	50.27 ± 24.99	49.74 ± 24.2	0.31
cFT (nmol/l)	0.26 ± 0.09	0.27 ± 0.12	0.26 ± 0.08	0.67
FTI	0.37 ± 0.15	0.37 ± 0.15	0.37 ± 0.15	0.56
TSI	3.30 ± 1.94	3.27 ± 1.95	3.3 ± 1.93	0.47
fPSA (mmol/l)	0.29 ± 0.52	0.38 ± 1.09	0.27 ± 0.25	<0.01
Glucose (mmol/l)	5.22 ± 1.47	4.98 ± 1.36	5.27 ± 1.49	0.49
Cholesterol (mmol/l)	5.38 ± 1.15	5.28 ± 1.14	5.37 ± 1.19	0.09
Triglyceride (mmol/l)	1.70 ± 1.75	1.89 ± 2.05	1.82 ± 1.8	0.01
HDL (mmol/l)	1.56 ± 0.53	1.64 ± 0.5	1.66 ± 0.52	0.80
Total protein (mmol/l)	80.94 ± 7.49	79.32 ± 6.3	81.24 ± 7.66	<0.01

BMI = body mass index; AC: abdomen circumference; AMS: aging males’ symptoms; TT = total testosterone; LH = luteinizing hormone; SHBG = sex hormone-binding globulin; cfT = calculated free testosterone; FTI = free testosterone index; TSI = testosterone secreting index; fPSA: free prostate specific antigen; HDL = high-density lipoprotein cholesterol; Statistical significance is set at p < 0.05.

The sex hormones levels of luteinizing hormone (LH), free testosterone (FT) and the testosterone secreting index (TSI) were not significantly different but total testosterone (TT) was significantly different (P = 0.02) between the vasectomy and non-vasectomy groups. The PSA and free PSA (fPSA) of the vasectomy group were higher than the non-vasectomy group. All the mean values from the blood biochemical tests were in the normal range, but there were significant differences in globin (*P* = 0.01) were found between the vasectomy subjects and the non-vasectomy subjects.

### Association between Vasectomy and Sex Hormones

The relationship between serum sex hormone levels and vasectomy was assessed by multiple linear regression analysis with the enter-model method (Table 2). The results revealed that vasectomy was not associated with the levels of TT, SHBG, cfT, FTI and LH after adjustments for Model 1 (age), Model 2 (model 1 + BMI + weight + AC) and Model 3 (model 2 + smoking and drinking status + education + marriage + residence). To evaluate the association between prostate cancer and vasectomy, PSA, fPSA and fPSA/PSA were also assessed via multiple linear regression analysis. The results showed that the relationship between prostate cancer and vasectomy was exceedingly weak and was not significantly different.

**Table 2:** Multivariate Linear Regression Analyses for serum sex hormone levels associated with vasectomy.

	Regression coefficient (95% CI)		
	Model 1	Model 2	Model 3
TT	0.25 (−0.29, 0.80)	0.27 (−0.27, 0.81)	0.07 (−0.48, 0.58)
SHBG	−0.004 (−0.05, 0.04)	−0.002 (−0.04, 0.04)	−0.02 (−0.06, 0.02)
LH	0.03 (−0.02, 0.08)	0.04 (−0.02, 0.09)	0.03 (−0.02, 0.08)
cfT	0.01 (−0.00, 0.02)	0.006 (−0.003, 0.02)	0.004 (−0.01, 0.01)
FTI	0.004 (−0.01, 0.02)	0.004 (−0.009, 0.02)	0.004 (−0.01, 0.02)
TSI	−0.03 (−0.21, 0.15)	−0.03 (−0.22, 0.15)	−0.07 (−0.25, 0.12)
PSA	0.08 (−0.03, 0.20)	0.09 (−0.02, 0.21)	0.09 (−0.21, 0.03)
fPSA	0.09 (−0.02, 0.20)	0.10 (−0.01, 0.21)	0.10 (−0.01, 0.21)
fPSA/PSA	0.001 (−0.009, 0.011)	0.001 (−0.009, 0.011)	0.002 (−0.008, 0.01)

TT = total testosterone; SHBG = sex hormone-binding globulin; LH = luteinizing hormone; cfT = calculated free testosterone.

FTI = free testosterone index; TSI = testosterone secreting index; PSA = prostate specific antigen; fPSA = free prostate specific antigen.

Predictors of model 1 = adjust age; Predictors of model 2 = predictors in model 1 + BMI + weight + abdomen circumference; Predictors of model 3 = predictors in model 2 + smoking and drinking status + education + married status + residence.

### Multivariate Linear Regression and Analyses of the Relationship between Vasectomy and the AMS and SF-36 scales

According to multiple linear regression analysis, vasectomy is not an independent variable in the models of the total AMS (Table 3). However, one of three aspects of AMS, the Somatic score was associated with vasectomy (0.31, 0.02 and 0.61). Health status was further tested with the SF-36 score and eight additional aspects. The results showed that vasectomy will significantly affect the SF-36 score in Model 4. “Role emotional” −6.28 (−10.34, −2.22) and “Mental health” −1.55 (−3.08, −0.02) were associated with vasectomy. Although these two psychological factors of the SF-36 were changed, vasectomy did not affect the Beck score.

**Table 3:** Multivariate Linear Regression Analyses for ageing males’ symptoms (AMS) scale associated with vasectomy.

	Regression coefficient (95% CI)			
	Model 1	Model 2	Model 3	Model 4
Somatic score	0.37 (−0.08, 0.82)	0.36 (−0.09, 0.81)	0.35 (−0.11, 0.79)	0.35 (−0.65, 0.08)
Psychological score	0.33 (0.04, 0.61)	0.32 (0.03, 0.61)	0.32 (0.02, 0.61)	0.31 (0.03, 0.61)
Sexual score	0.12 (0.30, 0.53)	0.06 (−0.35, 0.47)	0.02 (−0.40, 0.44)	0.02 (−0.58, 0.10)
Total AMS score	0.81 (−0.15, 1.78)	0.74 (−0.23, 1.71)	0.70 (−0.30, 1.64)	0.69 (−1.29, 0.29)
IPSS	0.24 (−0.71, 1.18)	0.21 (−0.74, 1.16)	0.31 (−0.68, 1.22)	−0.53 (−1.11, 0.41)
Beck score	0.516 (0.036, 0.997)	0.48 (−0.001, 0.96)	0.461 (−0.023, 0.946)	0.481 (−0.003, 0.965)
SF-36 score	−19.69 (−32.79, −6.59)	−18.90 (−32.05, −5.77)	−18.81 (−31.55, −5.10)	−18.8 (−32.00, −5.60)
Physical functioning	−1.50 (−3.69, 0.68)	−1.41 (−3.60, 0.79)	−1.69 (−3.82, 0.60)	−1.71 (−3.92, 0.50)
Role physical	−5.72 (−10.00, 1.44)	−5.14 (−9.44, −0.85)	−5.27 (−9.54, 0.88)	−5.24 (−9.57, 0.91)
Bodily pain	−1.94 (−3.75, −0.13)	−1.76 (−3.58, 0.05)	−1.57 (−3.32, 0.32)	−1.53 (−3.35, 0.30)
General health	−1.15 (−3.28, 0.99)	−1.11 (−3.25, 1.03)	−1.38 (−3.42, 0.88)	−1.40 (−3.55, 0.75)
Vitality	−0.81 (−2.43, 0.82)	−0.88 (−2.51, 0.74)	−0.60 (−2.18, 1.09)	−0.55 (−2.18, 1.09)
Social functioning	−0.53 (−1.78, 0.73)	−0.47 (−1.74, 0.80)	−0.55 (−1.77, 0.78)	−0.54 (−1.81, 0.74)
Role emotional	−6.48 (−10.50, −2.47)	−6.28 (−10.31, −2.25)	−6.12 (−10.18, −2.06)	−6.28 (−10.34, −2.22)
Mental health	−1.57 (−3.09, −0.05)	−1.85 (−3.37, −0.33)	−1.65 (−3.10, −0.05)	−1.55 (−3.08, −0.02)

Predictors of model 1 = adjust age; Predictors of model 2 = predictors in model 1 + BMI + weight + abdomen circumference + smoking and drinking status + education + married status + residence; Predictors of model 3 = predictors in model 2 + TT + SHBG + LH.

To lend further insight into the involvement of the scales and vasectomy, we determined the positive and negative values according to the score of the scales. The sample was divided into two groups by the thresholds of AMS = 27 or IPSS = 7. According to multiple logistic regression analysis vasectomy is not an effect factor for the AMS scales (*P* = 0.39, 1.10 (0.89, 1.35)). There is also no relationship between vasectomy and IPSS (*P* = 0.17, 1.21 (0.92, 1.58)).

### Multivariate Linear Regression Analyses of the Relationship between Vasectomy and Blood Biochemistry

To evaluate the effects of vasectomy on metabolism, as shown in Table 4, eight index values were tested and analyzed by multivariate analysis after adjustments for Model 1 (age), Model 2 (Model 1 + BMI + weight + AC), Model 3 (Model 2 + smoking and drinking status + education + marriage + residence) and Model 4 (Model 3 + TT + SHBG + LH). All models indicated that the indexes, including cholesterol, total protein, albumin, high-density lipoprotein, and globulin ratio are not significantly associated with vasectomy.

**Table 4:** Multivariate Linear Regression Analyses for blood biochemical test associated with vasectomy.

	Regression coefficient (95% CI)			
	Model 1	Model 2	Model 3	Model 4
Triglyceride	−0.12 (−0.31, 0.073)	−0.08 (−0.27, 0.11)	−0.08 (−0.27, 0.11)	−0.09 (−0.03, 0.25)
Cholesterol	0.05 (−0.07, 0.18)	0.056 (−0.07, 0.18)	0.07 (−0.06, 0.19)	0.067 (−0.14, 0.05)
Total protein	1.87 (0.95, 2.80)	1.85 (0.93, 2.78)	1.90 (0.94, 2.78)	1.837 (−0.64, 0.75)
Albumin	1.02 (0.45, 1.60)	1.05 (0.47, 1.63)	1.077 (0.46, 1.62)	1.03 (−0.06, 0.21)
High-density Lipoprotein	0.03 (−0.03, 0.09)	0.03 (−0.02, 0.09)	0.05 (−0.01, 0.10)	0.04 (−0.09, 0.01)
Globulin ratio	0.01 (−0.08, 0.09)	0.01 (−0.08, 0.09)	0.003 (−0.08, 0.09)	0.002 (−0.10, 0.02)
Glucose	0.29 (−0.08, −0.001)	0.28 (0.05, 0.51)	0.26 (0.029, 0.49)	0.25 (−0.20, 0.16)

Predictors of model 1 = adjust age; Predictors of model 2 = predictors in model 1 + BMI + weight + abdomen circumference + smoking and drinking status + education + married status + residence; Predictors of model 3 = predictors in model 2 + TT + SHBG + LH.

### Multivariate Linear Regression Analyses, Stratified by Selected Characteristics

As shown in Table 5, stratified analyses show that there are associations between vasectomy and the health scale. Four significant significantly affected factors by vasectomy included “Somatic score”, “SF-36”, “Role emotional” and “Mental health”, as determined by stratified analyses with Model 4. This analysis suggests that males aged 60 or older might have an increased risk for abnormal health compared to younger males. Men who smoke or drink may also have increased risk of these four factors. More changes were found with residence in township or more education, and this trend can significantly influence the four factors.

**Table 5:** Multivariate Linear Regression Analyses, Stratified by Selected Characteristics.

Stratification Variable	Psychological symptom	*P*	SF-36	*P*	Role emotional	*P*	Mental health	*P*
	Regression coefficient (95% CI)		Regression coefficient (95% CI)		Regression coefficient (95% CI)		Regression coefficient (95% CI)	
Age		0.165		0.003		0.001		0.046
40–59	0.22 (−0.17, 0.62)		−17.24 (−34.78, 0.30)		−5.18 (−10.63, 0.27)		−0.82 (−2.89, 1.24)	
>=60	0.37 (−0.07, 0.81)		−17.79 (−37.83, 2.26)		−7.01 (−13.11, −0.91)		−1.80 (−4.09, 0.49)	
BMI		0.161		0.002		0.001		0.045
<25	0.53 (0.16, 0.90)		−23.67 (−40.70, −6.64)		−12.33 (−38.67, 14.02)		−2.09 (−4.07, −0.12)	
>=25	−0.013 (−0.49, 0.46)		−12.00 (−32.99, 8.97)		−6.64 (−13.14, −0.14)		−0.75 (−3.20, 1.71)	
Smoker		0.154		0.003		0.001		0.048
no	0.27 (−0.18, 0.72)		−19.25 (−39.16, 0.66)		−6.21 (−11.47, −0.94)		−2.73 (−5.06, −0.40)	
yes	0.32 (−0.07, 0.71)		−19.42 (−37.17, −1.67)		−9.07 (−14.47, −3.68)		−0.76 (−2.80, 1.28)	
Drink		0.136		0.003		0.001		0.05
no	−0.90 (−0.59, 0.42)		−10.44 (−33.62, 12.74)		−2.46 (−9.43, 4.52)		−0.65 (−3.35, 2.05)	
yes	0.55 (0.19, 0.91)		−23.93 (−40.10, −7.77)		−8.37 (−13.40, −3.33)		−20.7 (−3.94, −0.21)	
Residence		0.162		0.002		0.001		0.048
village	0.23 (−0.47, 0.98)		−14.33 (−37.04, 9.54)		−6.23 (−29.51, −5.58)		−2.49 (−3.76, −1.59)	
township	0.35 (−0.25, 0.76)		−13.67 (−33.87, 5.22)		−7.67 (−13.75, −3.40)		−1.61 (−4.18, 1.33)	
Education		0.166		0.003		0.001		0.039
0–9 years	0.50 (0.05, 0.95)		−14.48 (−34.82, 5.85)		−4.18 (−10.34, 1.99)		−2.18 (−4.56, 0.21)	
>=10 years	0.19 (−0.20, 0.58)		−22.44 (−39.73, −5.14)		−8.06 (−13.44, −2.67)		−0.91 (−2.90, 1.08)	

### Prevalence of Symptoms Related to Vasectomy

Items that were significantly associated with vasectomy were selected. As shown in Table 6, the ordinal responses to the selected questions were then divided into symptomatic and asymptomatic categories. The Mann-Whitney test was used to confirm the differences in testosterone levels between the symptomatic group and the asymptomatic group. The three questions for “Role emotional” were significantly different between the two groups. There were no differences for the other three factors between the symptomatic and the asymptomatic group.

**Table 6:** Prevalence of Symptoms Related to Vasectomy.

Question Regarding Symptom	Evaluation Tool	Symptomatic	Asymptomatic	Symptom prevalence	Symptomatic Men With vasectomy
**Psychological symptom**					
Irritability (feeling aggressive, easily upset about little things, moody)	AMS	Moderate, Severe or Extremely severe	None or mild	13.9%	1.27 (0.96, 1.69)
Nervousness (inner tension, restlessness, feeling fidgety)	AMS	Moderate, Severe or Extremely severe	None or mild	15.6%	1.16 (0.84, 1.61)
Anxiety (feeling panicky)	AMS	Moderate, Severe or Extremely severe	None or mild	7.3%	1.13 (0.78, 1.66)
Depressive mood (feeling down, sad, on verge of tears, lack of drive, mood swings, feeling nothing is of any use)	AMS	Moderate, Severe or Extremely severe	None or mild	9.2%	1.02 (0.72, 1.44)
Feeling burnt out, having hit rock-bottom	AMS	Moderate, Severe or Extremely severe	None or mild	11.7%	1.00 (0.73, 1.37)
**Role emotional**					
Cut down the amount of time you spent on work or other activities	SF-36	no	yes	23.8%	0.73 (0.58, 0.91)
Accomplished less than you would like	SF-36	no	yes	25.4%	0.72 (0.58, 0.90)
Didn’t do work or other activities as carefully as usual	SF-36	no	yes	26.1%	0.73 (0.58, 0.91)
**Mental health**					
Have you been a very nervous person?	SF-36	All or most of the time	Sometimes, a little, or none of the time	46.8%	1.03 (0.84, 1.27)
Have you felt so down in the dumps that nothing could cheer you up?	SF-36	All or most of the time	Sometimes, a little, or none of the time	62.6%	1.17 (0.95, 1.44)
Have you felt calm and peaceful?	SF-36	All or most of the time	Sometimes, a little, or none of the time	9.8%	0.84 (0.60, 1.16)
Have you felt downhearted and blue?	SF-36	All or most of the time	Sometimes, a little, or none of the time	60.9%	1.00 (0.81, 1.24)
Have you been a happy person?	SF-36	Sometimes, a little, or none of the time	All or most of the time	10.0%	1.18 (0.85, 1.64)

## Discussion

Our study explored the effects of vasectomy through many aspects, and we confirmed that men with vasectomy for over 15 years will not have extended physiological problems, including sex hormones, cancer risk, or physiological rank. However, our results suggest that vasectomy may have psychological effects, such as “psychological symptom, role emotional and mental health”.

We first focused on the level of testosterone after vasectomy. Some researchers found that these symptoms were the result of low androgen levels^16,17^. In studies of rats, rabbits and monkeys when spermatogenesis was damaged or normal, serum testosterone levels were significantly reduced^18^. In this study, we did not find a significant change in the total testosterone or free testosterone though a multivariate linear model and the testosterone levels are all in the norm range. These results agree with Xiang’s research, which found no significant difference in testicular or epididymal size via qualitative histology after vasectomy^19^. Although evidence regarding hormone levels has been conflicting, there is a general consensus that they are within the normal range following vasectomy^20^. Also we tested FTI and TSI and inferred that the ability of the testis is unaffected after a long-term vasectomy. These results suggest that sperm production and sperm storage/removal might remain balanced after long-term epididymal distension. It is noteworthy that male reproductive hormonal levels may depend on the ethnic differences^21^. However, we did not analyze the factor that all the participants are East Asian, including a 98.5% (2388/2425) Han Chinese population in this study.

Late-onset hypogonadism (LOH) is a common disorder in older men, but it is often underdiagnosed and untreated^22,23^. After examining testosterone, we assessed the AMS and found no change in the AMS level. This result suggested that the vasectomy will not increase the risk of LOH. To explore the association between vasectomy and a risk of prostate cancer. By testing the PSA level, some researchers reported an increased prostate cancer risk after vasectomy, especially during a 24-year follow-up study^24^. However, many other studies and meta-analyses^25^ found no association between vasectomy and non-vasectomy groups. In our study, we found that evidence of a relationship between prostate cancer risk and vasectomy was not established. A limitation is that we did not perform a digital rectal examination, ultrasound or prostatic biopsy to diagnose prostate cancer.

Currently, research on vasectomy leading to long-term psychological effects is relatively scarce. Ehn. *et al*.^26^ evaluated the long-term satisfaction of 108 vasectomized men through a mailed questionnaire 2 and 7 years after the operation. Through retrospective analysis, they found that vasectomy had no major effects on the physical health of men. Another study revealed that sterilization psychologically affected depressive symptoms and anxiety^27^. Therefore, long-term quality of life assessment and mental status after vasectomy were our key focus. Our results showed that middle-aged and older people are mainly influenced at the psychological level rather than the physiological level after vasectomy. These results may be related to the following factors: First, vasectomy failure occurs in 0–2% of patients^28^. People were warned that although reversal is feasible, it is not invariably successful. They could be afraid that the procedure may have to be repeated if there are persistent motile sperm in their post-vasectomy semen analysis^29^. Second, the etiology of post-vasectomy pain syndrome is not clear and pain after vasectomy is a challenging male urological problem. There is no reliable effective treatment, which further frustrates both the patient and clinician because many of these patients will end up seeing physicians across many disciplines^30^. Third, from the 1970s to 1990s, the Chinese government proposed a population and family planning policy in which the state adopted a series of sterilization measures, including the vasectomy of men with two children. Some of these men received an involuntary vasectomy, and they were not provided preoperative consultation according the vasectomy guideline and policy at that time. They view vasectomy as a symbolic castration, which has led to fears about masculinity, male identity, and the loss of living children. These changes resulted in an adverse effect on psychological adjustment in some men that triggered the onset of depression and anxiety^31,32^. This outcome may led to a series of psychological problems, while we dot have the information of medical record or specialized examination to support the result.

In addition, potential confounders in the association between men with vasectomy and men without vasectomy have rarely been studied. Our stratified analyses indicated that the SF-36 score and “role emotional” were related to the subject’s age, BMI index, cigarette smoking and drinking status, and educational level. Men after vasectomy of an older age tended to be more susceptible to complications. Mental state and quality of life are easily affected if the men enjoy smoking and drinking. Moreover, stratified analyses indicated that a higher degree of education was inversely correlated with life satisfaction. It is important to note that in this article the overall education level was relatively low. We found that 41.4% of the participant’s education levels were lower than 9 years. We inferred that people with a higher education level would have anxiety because of worry about the success rate, complications, chronic post-operative pain, altered family life and dissatisfaction with the government’s family planning policy. Subahani *et al*. reported that these complications have generally occurred at higher rates in developing countries, and are linked to limited knowledge about the procedure and inadequate pre-operative counseling. In contrast, encouragement, proper information and good marital relations increased the likelihood of having the procedure performed^33^.

Consequently, an urgent problem that should be solved is how to reduce psychological problems after vasectomy. In our study, we evaluated the prevalence of symptoms related to vasectomy. A significant difference occurred for role emotional. Psychological distress appears to be due to poor pre-operative counseling on the vasectomy, such as the patients can not get information of the specific type of procedure and anesthesia. In developing countries in particular, there is a limited understanding for vasectomy and negative attitudes have been linked to anxiety about vasectomy^34,35^. Some participator with vasectomy maybe are excessive fear of a surgical procedure, and they should be record in the pre-operation. The person with Notably our result did not indicate that the vasectomy will cause neurological disorders in the long time. Additionally, the inflammatory and immunological consequences are potential negative effects of a vasectomy. Though some studies explored the inflammatory biomarkers, but there were no sensitive or very specific evidences^36^. We should explore the reliable biomarkers in the future study.

In conclusion, proper and careful pre-operative counseling is indispensable before vasectomy. Informing men of the safety and the high success rate of vasectomy, as well as the possible immediate and long-term complications, will help to eliminate their anxiety and fear. Meanwhile, psychological counseling for smokers, drinkers, the elderly, and those who underweight individuals are particularly important. They should be counseled that vasectomy will not affect their work efficiency, which should eliminate their doubts in the role emotion questionnaire.

## Materials and Methods

### Study population

In this study, 4091 community-dwelling Chinese adult men who were aged more than 40 years-old in 3 areas of the Beijing, Hubei and Jiangsu provinces of China were recruited at the local reproductive health services clinic. The trained nurses and investigators introduced the study and after signing an informed consent the participants were invited to complete interviewer-assisted questionnaires, undergo a general physical examination and receive a blood draw for testing. We screened 542 men with a history of no-scalpel vasectomy, then we excluded 27 men because the time since they had their vasectomy was less than 15 years. Among the 515 men, a total of 30 men were excluded for missing demographic information (8 men), missing blood biochemistry test data (13 men) or missing questionnaire data (9 men). The remaining 485 men (154 men from Beijing, 191 men from Hubei and 140 men from Jiangsu respectively) with a history of vasectomy (>15 years) were used as the vasectomy group, and 1940 men (625 men from Beijing, 785 men from Hubei and 530 men from Jiangsu respectively) of the 3549 men without vasectomy were chosen by 1:4 random match according to cluster and age stratified sampling.

The study was approved by the Ethical Committee Review Board of Tongji Medical College, Huazhong University of Science and Technology, China. The experimental protocols were performed according to the approved guidelines. Written informed consents was obtained from all enrolled participants.

### Basic information

All participants completed the tables for basic information including age, education level, marital status and residence. Smoking status was classified as never smoked, former smoker and current smoker. Alcohol consumption was classified according to the frequency of alcohol intake, including beer, wine and white spirits, per week. During the health screening, the results of the physical examination were recorded including height, weight, body mass index (BMI), and abdomen circumference. BMI (kg/m^2^) = [body weight of subject] (kg)/[square of height of subject] (m^2^).

### Questionnaires

Three questionnaires (Chinese version), including the AMS, IPSS and SF-36, were completed with interviewer-assistance. The AMS questionnaire included 17 symptoms divided into three groups (psychological, sexual and somato vegetative) and assessed the intensity of andropause symptoms. The International Prostate Symptom Score(IPSS) included three questions regarding filling problems and four questions regarding voiding problems were used to assess prostate symptoms. The SF-36 questionnaire included eight areas (physical functioning, role physical, bodily pain, general health, vitality, social functioning, emotional role, and mental health) and was used to assess the quality of life.

### Hormone assays

Blood samples were collected between 08:00 am and 10:00 am. All serum samples were measured in batches in the laboratory center of Wuhan Tongji Reproductive Medicine Hospital. Serum TT, sex hormone-binding globulin (SHBG) and luteinizing hormone (LH) were assayed by chemiluminescent immunoassays on a Beckman Access Immunoassay System (Beckman Coulter, USA). cFT was calculated from TT and SHBG using mass action equations as described by a previous study^18^. Testosterone secreting Index (TSI) was calculated using the TT/LH equation and free testosterone index (FTI) was calculated using the TT/SHBG equation. Serum concentrations of fasting blood glucose (FBG), triglycerides (TG) and high-density lipoprotein cholesterol (HDL-C) were measured directly with a Roche combas6000 System (Hoffmann-La Roche Ltd., Sweden).

### Blood biochemical test and PSA test

Fasting plasma glucose levels, fasting insulin and serum levels of triglycerides, total cholesterol, total protein, albumin, high-density lipoprotein, and the globulin ratio were measured with a Cobas c311 clinical chemistry analyzer (Hoffmann-La Roche Ltd., Sweden). Reference values were from the SRL Test Dictionary 2004.35. Fasting plasma glucose (fasting blood glucose, FBG) <3.9 mmol/L as hypoglycemia, 3.9–6.1 mmol/L was normal, and >6.1 mmol/L was hyperglycemic. Cholesterol (CHO) <5.72 mmol/L was normal, and ≥5.72 mmol/L was high cholesterol. Triglycerides (triglyceride, TG) at 0.55–1.70 mmol/L was normal, and ≥1.70 mmol/L was high triglycerides. HDL (high density lipoprotein, HDL) ≤0.91 mmol/L was reduced, and 0.91–2.07 mmol/L was normal. PSA was detected by a chemiluminescence immunoassay using an Access 2 Immunoassay System (Beckman Coulter Co., Ltd.).

### Statistical analyses

Statistical analyses were performed using SPSS statistical software (version 19.0, SPSS Inc, Chicago, IL). Continuous variables were represented as the mean ± standard deviation (SD) and categorical data were represented by number (n) and percentage (%). Comparisons between the two groups were tested using the independent two sample t-test for continuous variables, and the chi-square test for categorical variables. We used a multivariate linear model with robust regression to associate vasectomy and other indexes. The indexes of TT, SHBG and LH were natural log transformed before a linear model analysis to ensure normality. Logistic regression analysis with a forward selection method was performed to analyze the vasectomy and health status questionnaire. Stratified analyses were performed for age (two categories: 40–59, ≥60 years), BMI (two categories: <25, ≥25 kg/m^2^), smoking (two categories: non-smoking, ever-smoking), drinking (two categories: non-drinker, ever-drinker) and education (two categories: 0–9, ≥10 years)to compare the association between vasectomy and mental health. All P values were 2-sided. A P value < 0.05 was considered statistically significant.
